# Identification of stroke biomarkers using proteomic profiling of extracellular vesicles derived from human blood: a preliminary study

**DOI:** 10.3389/fneur.2025.1587389

**Published:** 2025-07-02

**Authors:** Tae Yeon Kim, Jae Yeon Park, Yun Seo Cho, Dong Hyuk Youn, Sung Woo Han, Harry Jung, Yong-Jun Cho, In cheol Jeong, Jin Pyeong Jeon, Kangeun Ko, Hyo Youl Moon

**Affiliations:** 1 Department of Physical Education, Seoul National University, Seoul, Republic of Korea; 2 Institute of New Frontier Research, Hallym University College of Medicine, Chuncheon, Republic of Korea; 3 Department of Neurosurgery, Hallym University College of Medicine, Chuncheon, Republic of Korea; 4 Department of Artificial Intelligence Convergence, Hallym University, Chuncheon, Republic of Korea; 5 Institute of Aging, Seoul National University, Seoul, Republic of Korea; 6 Learning Sciences Research Institute, Seoul National University, Seoul, Republic of Korea

**Keywords:** hemorrhagic stroke, ischemic stroke, extracellular vesicles, biomarker, proteomics

## Abstract

**Introduction:**

Stroke is a devastating brain disease that causes extensive neurological impairment and high mortality. Rapid diagnosis and intervention of stroke are necessary to minimize neurological damage and improve recovery. Extracellular vesicles (EVs) have been identified as potential biomarkers for stroke, suggesting promising avenues for rapid diagnosis and prognostic assessments.

**Methods:**

This preliminary study aimed to evaluate the potential of EVs as biomarkers in the distinct pathophysiological mechanisms of hemorrhagic stroke (HS) and ischemic stroke (IS). We have identified proteins differentially expressed in EVs derived from the blood of HS and IS patients. EVs were isolated using an isolation kit, followed by proteomic analysis by LC-MS/MS to compare protein expression patterns.

**Results:**

As a result, 15 proteins were upregulated and 4 downregulated in HS-derived EVs, and 14 proteins were upregulated and 5 downregulated in IS-derived EVs. Among these, four proteins were commonly upregulated and one protein was commonly downregulated in both stroke types, whereas the remaining proteins exhibited stroke-type-specific expression patterns. To further explore the proteomic findings, we confirmed the increased levels of CRP and PF4 in HS patients using ELISA, verifying their elevation in patient blood samples.

**Discussion:**

Although this additional evaluation was conducted only for HS, these findings suggest that EV-derived proteins have potential as biomarkers for both HS and IS, supporting their broader applicability in stroke diagnosis.

## 1 Introduction

Stroke is one of the leading causes of death and disability worldwide, posing a significant public health challenge (1). It is classified into two main types: hemorrhagic stroke (HS) and ischemic stroke (IS). HS occurs due to the rupture of blood vessels, whereas IS is caused by the obstruction of blood vessels due to a thrombus. Rapid identification and timely treatment of stroke are essential to minimize neurological damage and enhance recovery, underscoring the critical importance of early diagnosis and effective prevention strategies (2). Stroke diagnosis is made through a physician’s clinical examination, based on a combination of blood tests and imaging techniques such as computerized tomography (CT) and magnetic resonance imaging (MRI). While current diagnostic tools are indispensable for managing stroke, they have limitations, including a lack of specificity, in addressing side effects and managing the short therapeutic window. Due to these limitations, there is an increased need for biomarkers that are sufficiently sensitive, specific, and rapid. Specifically, distinct markers are needed to indicate the different pathogenic mechanisms of HS and IS.

Extracellular vesicles (EVs) are involved in intercellular communication by transporting biomolecules such as proteins, lipids, and RNAs. Their lipid bilayer encapsulation protects these contents from degradation, enabling precise analytical assessments (3). EVs encapsulate molecular components representative of their originating cells, allowing them to capture pathological changes and offering significant potential as tools for identifying disease-specific biomarkers (4). Among the various components of EVs, protein analysis plays a pivotal role in biomarker research for a wide range of diseases such as cancer (5), cardiovascular diseases (6), and neurodegenerative diseases (7). This is because proteins reflect the pathological state of the disease and provide valuable insights for both diagnosis and prognosis. In the case of stroke, proteomics emerges as an especially powerful tool. By identifying specific proteins linked to stroke, researchers can uncover biomarkers critical for accurate diagnosis and prognosis. Compared to other analytical methods, proteomics offers a broader and more precise analysis, enabling researchers to explore the mechanisms of stroke and examine variations across different stages of the disease based on protein expression patterns (8). This level of detailed insight into protein changes offers a unique advantage, particularly in studying complex conditions like stroke, where multiple factors influence disease progression and outcomes.

This pilot study aims to identify differential proteins in blood-derived EVs between control subjects and patients with HS and IS, to assess the potential of EVs as biomarkers for HS and IS. To support the clinical relevance of the identified candidates, we conducted ELISA-based analysis of selected proteins (CRP and PF4) in the plasma of HS patients.

## 2 Materials and methods

### 2.1 Subjects and sample collection

Blood samples for the control group were collected from Seoul National University, while the patient cohort was recruited from Chuncheon Sacred Heart Hospital. Patients were enrolled if their clinical diagnoses and neuroimaging findings were consistent with either hemorrhagic or ischemic stroke. The inclusion and exclusion criteria for both control and patient groups are summarized in Supplementary Table 1. This study was approved by the Institutional Review Board of Seoul National University (No. 2401/002–003) and Hallym University Chuncheon Sacred Heart Hospital (No. 2018–01-006). The characteristics of the participants are shown in Tables 1, 2.

**Table 1:** Participants characterization for proteomic analysis.

No.	Con (*n* = 10)	HS (*n* = 4)	IS (*n* = 6)	*p*-value
Mean age (years)	26.9 ± 1.5	66.0 ± 20^a^	80.7 ± 11.4^a^	<0.0001
Sex ratio (female: male) (%)	50.0: 50.0	75.0: 25.0	16.7: 83.3	0.2034
Days from stroke onset	0	2.8 ± 1.0	1.8 ± 0.4	
Systolic blood pressure (mmHg)	121.2 ± 5.6	132.5 ± 6.6	129.3 ± 15.1	0.1066
Diastolic blood pressure (mmHg)	71.7 ± 8.1	83.3 ± 19.0	77.7 ± 12.0	0.2604

Statistical comparisons between control and stroke patient groups were performed using one-way ANOVA with a Bonferroni *post hoc* test for continuous variables (age, days from stroke onset, systolic blood pressure, diastolic blood pressure) and Chi-square tests for categorical variables (sex). A *p*-value < 0.05 was considered statistically significant. (a) indicates *p* < 0.05 vs. control.

**Table 2:** Participants characterization for ELISA.

No.	Con (*n* = 16)	HS (*n* = 32)	*p*-value
Mean age (years)	34.4 ± 11.4	42.9 ± 7.9	0.0044
Sex ratio (female: male) (%)	56.3: 43.8	53.1: 46.9	>0.9999
Days from stroke onset	0	2.5 ± 1.7	<0.0001

Statistical comparisons between control and stroke patient groups were performed using independent two-tailed *t*-tests for continuous variables (age and days from stroke onset) and Chi-square tests for categorical variables (sex). A *p*-value < 0.05 was considered statistically significant.

### 2.2 EVs isolation from plasma samples

Blood was collected from both stroke patients and control subjects to obtain plasma, and exosomes were then isolated using the ExoQuick™ kit (System Biosciences Inc., CA, United States), according to the manufacturer’s instructions. Briefly, blood samples were collected in EDTA tubes and centrifuged at 1,000 × *g* for 10 min at 4°C. The supernatant was transferred to a 15 mL conical tube and centrifuged again at 2000 × g for 15 min at 4°C. The resulting supernatant was transferred to another conical tube, mixed with an equal volume of phosphate-buffered saline (PBS), and centrifuged at 4,500 × *g* for 20 min at 4°C. ExoQuick reagent was then added to the supernatant, and the mixture was incubated at 4°C for 2 h. Following this, the samples were centrifuged at 1,500 × *g* for 30 min at 4°C, retaining the pellet. The pellet was then centrifuged again at 1,500 × *g* for 5 min at 4°C, and the supernatant was carefully removed. The pellets were resuspended in PBS and used for Nanoparticle tracking analysis (NTA) and proteomic analyses.

### 2.3 Identification of EV characterization

Extracellular vesicles size was analyzed using the NanoSight NS300 (Malvern Panalytical Ltd., Malvern, UK) NTA system. For all NTA experiments, each sample was measured in five repeated runs with 60-s intervals, and 1X-filtered PBS was used as an absolute control to evaluate the purity of the resuspension medium. Each sample was diluted 500-fold to ensure it fell within the operational range of the NS300 instrument (1 × 10^7^–10^9^ particles/mL).

### 2.4 Proteomic analysis

#### 2.4.1 Filter aided sample preparation (FASP) digestion

Protein concentrations were determined using the bicinchoninic acid (BCA) assay, and approximately 100 μg of protein per sample was subjected to filter-aided sample preparation (FASP) as previously described (9). For reduction, samples were treated with 5 mM TCEP at 33°C for 30 min, followed by centrifugation at 14,000 × *g* for 15 min. Alkylation was then performed using 50 mM iodoacetamide (IAA) at 25°C in the dark for 30 min, after which the samples were centrifuged under the same conditions. The filters were washed three times with 8 M urea in 0.1 M Tris–HCl (pH 8.5) and an additional three times with 50 mM ammonium bicarbonate (ABC). Proteins were digested with trypsin at a 1:50 enzyme-to-protein ratio in 50 mM ABC buffer, and incubated at 37°C for 12 h. Peptides were recovered by centrifugation and dried using a vacuum concentrator. Prior to desalting, peptide concentrations were estimated to be approximately 1 μg/μL.

#### 2.4.2 Desalting

Peptides were desalted using Micro C18 Spin-Columns with 75 μL of 80% acetonitrile (in 0.1% Trifluoroacetic acid). After desalting, the eluate was dried using a Speed-Vac and stored at −20°C until further analysis.

#### 2.4.3 Liquid chromatography-mass spectrometry/mass spectrometry (LC–MS/MS) analysis

Peptide separation and identification were performed using an UltiMate 3,000 RSLC nano LC system (Thermo Fisher Scientific) coupled to a Q Exactive mass spectrometer (Thermo Fisher Scientific). Peptide samples were first loaded onto a C18 trapping column (3 μm, 100 Å, 75 μm × 2 cm), followed by separation on an analytical column (PepMap™ RSLC C18, 2 μm, 100 Å, 75 μm × 50 cm). The mobile phases consisted of solvent A (water with 0.1% formic acid) and solvent B (80% acetonitrile with 0.1% formic acid). The mass spectrometer was operated in data-dependent mode with a mass range of 400–2000 m/z. Raw MS/MS data were processed using Proteome Discoverer™ software (version 2.5), and database searches were performed against the UniProt human (*Homo sapiens*) proteome using the SEQUEST HT search engine. Detailed quantification and filtering criteria are described in Section 2.5.

### 2.5 Data analysis of the proteome

Label-free quantification was performed using Proteome Discoverer™ software (ver.3.1). The *Homo sapiens* database from UniProt was used for protein identification. The analysis workflow included recalibration and calculation of precursor masses through Spectrum Files RC, and protein identification was performed using the SEQUEST HT algorithm for database searches. The quantification spectra used to determine abundance values were carried out using the precursor ions quantifier. Precursor abundance calculations were based on intensity.

The search parameters included a precursor ion mass tolerance of 10 ppm, a fragment ion mass tolerance of 0.02 Da, and a maximum of two missed cleavages using trypsin. The dynamic modifications applied to the peptide sequences were as follows: static carbamidomethylation of cysteine (+57.012 Da), variable oxidation of methionine oxidation (+15.995 Da), N-terminal acetylation (+42.011 Da), and N-terminal carbamylation (+43.0006 Da).

Protein identification was filtered using a false discovery rate (FDR) threshold of <1% based on a target-decoy strategy. Only proteins with high-confidence identifications, including at least one unique peptide and a minimum peptide length of six amino acids, were retained. Protein quantification was normalized using the “total peptide amount” method. Fold changes were calculated as the ratio of the mean normalized protein abundance in the stroke group to that in the control group. Statistical significance was determined using a two-sided Student’s t-test based on the normalized abundance values of individual samples.

### 2.6 Enzyme-linked immunosorbent assay (ELISA)

We centrifuged the whole blood at 1,000 × *g* for 10 min to isolate the plasma. To further eliminate residual platelets, an additional centrifugation step at the same speed and duration was performed at 2–8°C. The ELISA kits were used: C-Reactive Protein (CRP) (1: 50, R&D Systems, MN, United States) and Platelet Factor 4 (PF4) (1: 10, R&D Systems, MN, United States). We followed the manufacturer’s instructions, with modifications made to the incubation times as appropriate. Specifically, incubation times for CRP and PF4 were set at 30 min. The plate was analyzed using a NanoQuant Infinite M200 spectrophotometer (Tecan Group Ltd., Männedorf, Switzerland).

### 2.7 Statistical analysis

Statistical analysis was performed using GraphPad Prism (GraphPad Software Inc., CA, United States). Comparisons between the control subjects and HS patients were analyzed using Student’s *t*-test. The data were given as the mean ± standard error (SEM). *p*-values < 0.05 were reported as statistically significant.

## 3 Results

### 3.1 Characterization of EVs

**Figure 1:**
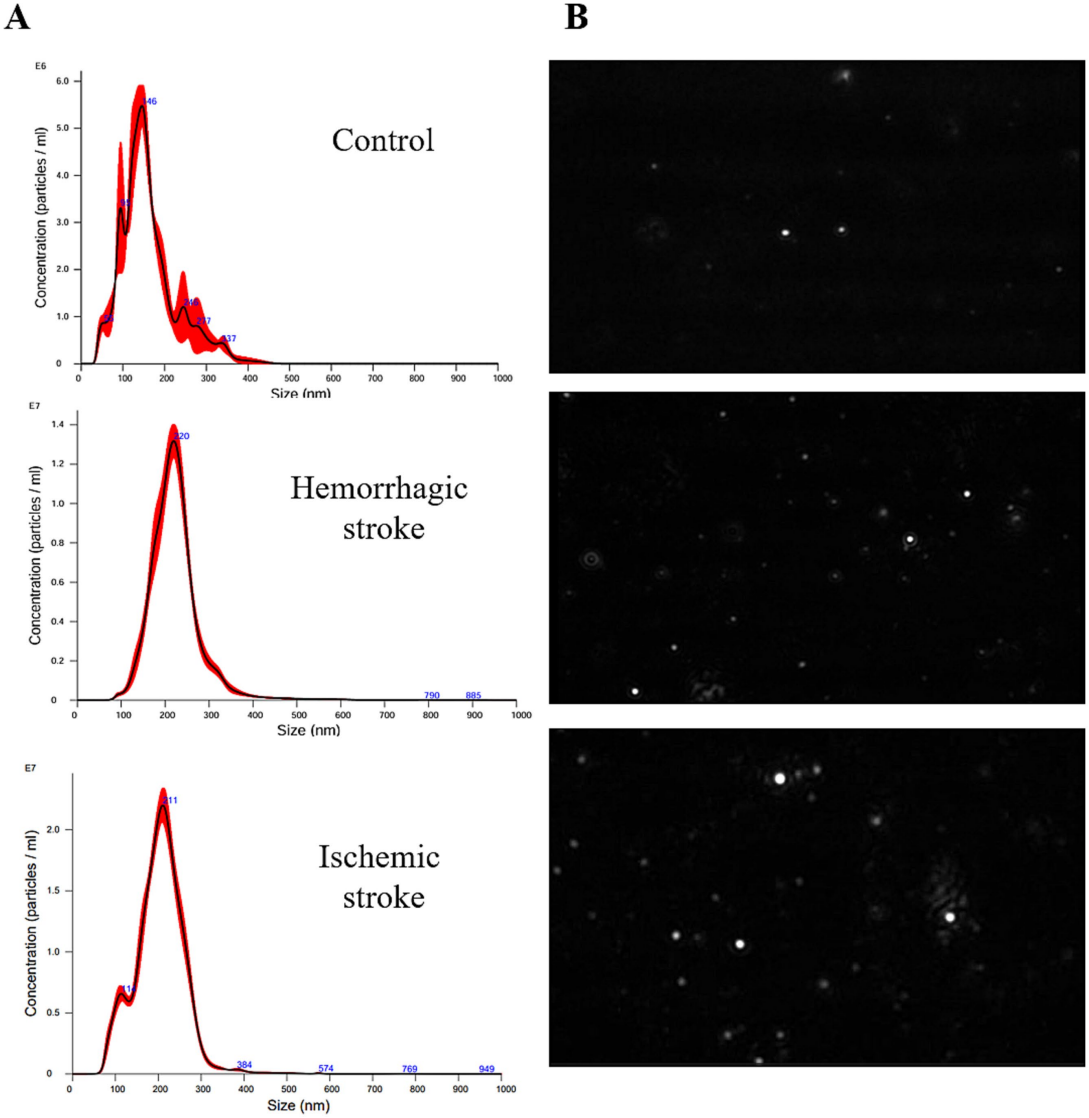
Size characterization of human blood-derived EVs by NTA. **(A)** Size distribution of EVs in control, HS, and IS. **(B)** NTA video frames of 0.3ug/mL EVs in stroke and control.

The NTA was performed to evaluate the size and concentration of EVs isolated from control subjects and stroke patients (Figure 1). The size range of EVs was 114 ~ 230 nm (average size is 143.8 ± 5.1 nm in the control group, 213.4 ± 2.5 nm in the HS group, and 201.1 ± 2.8 nm in the IS group). Particle concentrations were detected as 1.24 × 10^9^ particles/mL in the control group, 1.42 × 10^9^ particles/mL in the HS group, and 3.37 × 10^9^ particles/mL in the IS group (Table 3).

**Table 3:** EVs characterization by NTA.

No.	Con		HS		IS	
	Size	Concentration	Size	Concentration	Size	Concentration
1	161.0 ± 7.9	6.03E+08	224.0 ± 2.9	1.35E+09	220.3 ± 2.5	2.09E+09
2	114.7 ± 4.1	1.41E+09	212.4 ± 1.6	1.42E+09	158.8 ± 1.7	7.16E+09
3	172.8 ± 2.4	4.69E+08	204.6 ± 2.4	1.15E+09	202.4 ± 4.3	8.11E+08
4	121.9 ± 4.9	1.35E+09	212.7 ± 3.2	1.78E+09	190.5 ± 1.5	7.12E+09
5	136.9 ± 2.2	3.58E+09			230.7 ± 4.4	5.06E+08
6	134.5 ± 9.0	1.90E+09			204.1 ± 2.1	2.58E+09
7	172.9 ± 3.8	5.19E+08				
8	146.6 ± 9.3	6.95E+08				
9	119.9 ± 3.6	1.25E+09				
10	156.9 ± 3.6	6.02E+08				

Size range: 114.7 ~ 230.7 nm.

Average size (nm): Control 143.8 ± 5.1 nm, Hemorrhage stroke 213.4 ± 2.5 nm, Ischemic stroke 201.1 ± 2.8 nm.

Average concentration (particles/ml): Control 1.24E+09, Hemorrhage stroke 1.42E+09, Ischemic stroke 3.37E+09.

Values for size represent the mean ± standard error of the mean (SEM).

### 3.2 Proteomic analysis of human blood-derived EV proteins in stroke and control groups

**Figure 2:**
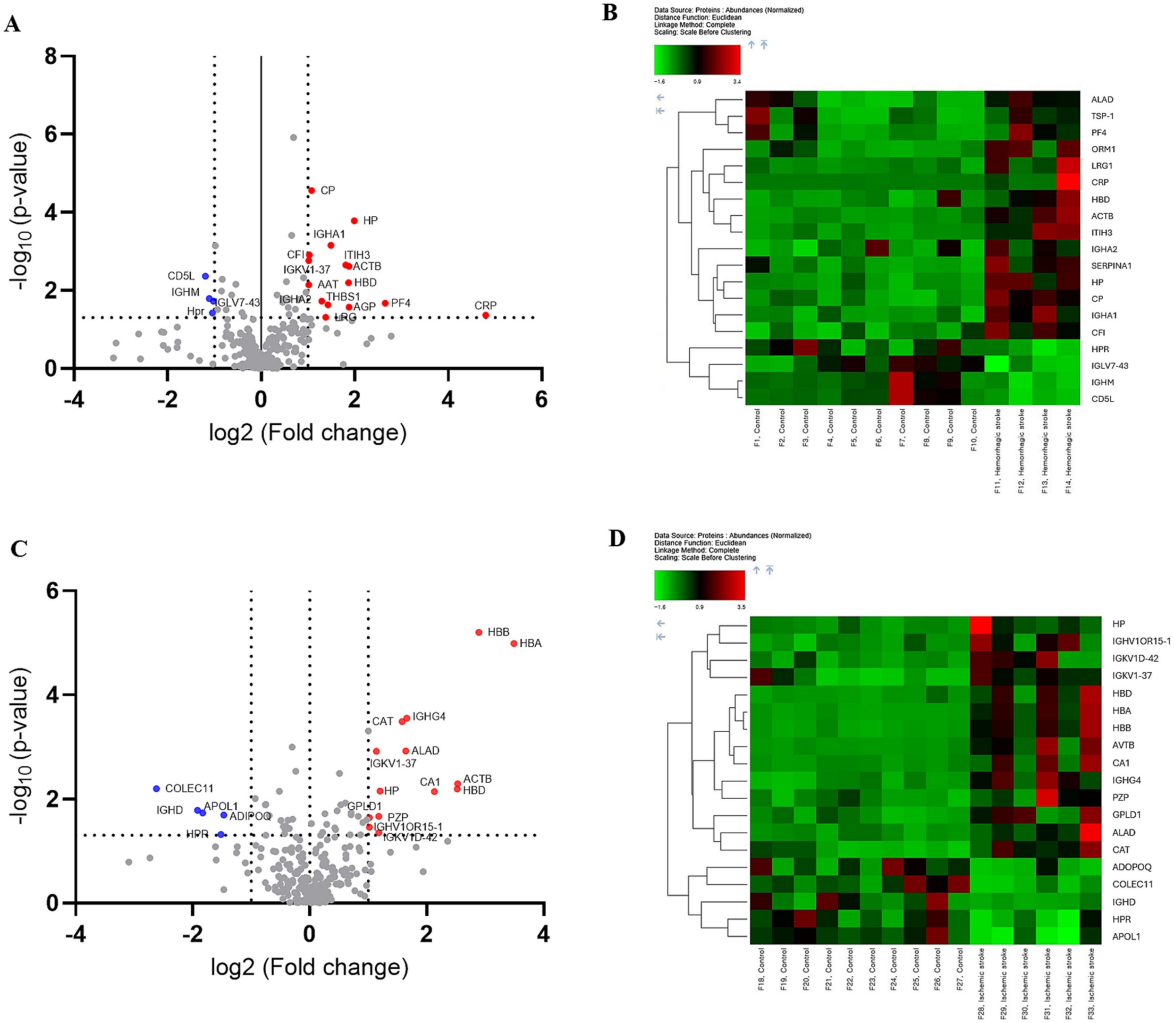
Proteomic analysis of human blood-derived EV proteins in controls versus hemorrhagic and ischemic stroke patients. Differential protein expression analysis: **(A,B)** Controls vs. hemorrhagic stroke patients. **(C,D)** Controls vs. ischemic stroke patients. **(A,C)** Volcano plot highlighting significantly upregulated and downregulated proteins, with a fold change threshold of 1 and *p*-value < 0.05. **(B,D)** The heat map depicts the expression patterns of proteins with differential expressions, with red indicating high expression and green indicating low expression.

To capture biomarkers specifically associated with the early pathophysiological responses to HS and IS, blood samples were collected at 2.4 ± 1.0 days post-onset (Table 1). Subsequently, LC–MS/MS proteomics analyses were performed on blood-derived EV from both HS and IS patients, identifying 29 distinct proteins (Supplementary Table 3). Given the divergent pathogenic mechanisms underlying HS and IS, we segregated the patient cohorts to investigate the unique protein expression profiles associated with each stroke subtype. This approach will enable targeted analysis of biomolecular alterations specific to the pathophysiology of HS and IS. Comparing HS patients and control groups, a total of 19 differentially expressed proteins (DEPs) were identified, of which 15 proteins were upregulated and 4 proteins were downregulated (Figure 2A). In addition, comparing IS and control groups, a total of 19 DEPs were identified, of which 14 proteins were upregulated and 5 proteins were downregulated (Figure 2C). Details of these DEPs are shown in Tables 4, 5. The heatmap, generated to identify specific EV protein expression patterns in each stroke group (Figures 2B,D), revealed distinct profiles that demonstrate clear separation between the control group and both HS and IS groups.

**Table 4:** Identification of differentially expressed proteins between the hemorrhagic stroke and control groups.

No.	Accession	Protein name (symbol)	Fold change (Stroke/Control)	*P*-value	Control mean	Control SD	Hemorrhagic stroke mean	Hemorrhagic stroke SD
1	A0A075B6R2	C-reactive protein (CRP)	4.8	0.04362	1.82e+06	1.48e+06	5.46e+05	5.62e+05
2	Q15848	Platelet factor 4 (PF4)	2.65	0.02129	6.24e+06	3.07e+06	4.58e+06	5.85e+06
3	O14791	Haptoglobin (HP)	1.99	0.00016	2.51e+07	6.07e+06	1.32e+07	4.98e+06
4	P00739	Inter-alpha-trypsin inhibitor heavy chain H3 (ITIH3)	1.88	0.00242	5.67e+07	2.36e+07	2.76e+07	1.12e+07
5	P04180	Alpha-1-acid glycoprotein 1 (ORM1)	1.88	0.02702	2.43e+05	1.50e+05	6.87e+04	7.44e+04
6	Q9BWP8	Hemoglobin subunit delta (HBD)	1.87	0.00633	3.41e+05	2.15e+05	1.08e+05	1.05e+05
7	O43866	Actin, cytoplasmic 1 (ACTB)	1.81	0.00224	2.12e+08	9.68e+07	7.75e+07	2.65e+07
8	A0A024R6I7	Immunoglobulin heavy constant alpha 1 (IGHA1)	1.49	0.00070	1.73e+05	7.75e+04	1.07e+06	1.72e+06
9	A0A075B6S9	Thrombospondin-1 (THBS1)	1.43	0.02357	2.46e+07	1.07e+07	3.97e+07	5.02e+06
10	P18428	Leucine-rich alpha-2-glycoprotein (LRG1)	1.38	0.04874	3.76e+06	1.29e+06	3.09e+07	3.97e+07
11	A0A0G2JMB2	Immunoglobulin heavy constant alpha 2 (Fragment) (IGHA2)	1.3	0.01897	8.21e+07	6.35e+07	1.50e+08	5.13e+07
12	A0A096LPE2	Ceruloplasmin (CP)	1.08	0.00003	3.22e+06	5.48e+05	6.40e+06	3.53e+06
13	P06702	Complement factor I (CFI)	1.03	0.00123	1.26e+05	6.95e+04	1.69e+05	1.54e+05
14	P20742	Alpha-1-antitrypsin (SERPINA1)	1.02	0.00719	1.83e+06	6.89e+05	5.96e+06	3.59e+06
15	P02763	Probable non-functional immunoglobulin kappa variable 1–37 (IGKV1-37)	1.02	0.00172	3.34e+06	1.55e+06	8.76e+06	3.44e+06
16	Q06033	Immunoglobulin lambda variable 7–43 (IGLV7-43)	−1.02	0.01880	1.03e+06	3.22e+05	4.24e+06	1.67e+06
17	P00738	Haptoglobin-related protein (HPR)	−1.04	0.03745	2.38e+08	9.00e+07	8.57e+08	2.14e+08
18	P07996	Immunoglobulin heavy constant mu (IGHM)	−1.11	0.01622	9.17e+06	8.58e+06	1.53e+07	5.10e+06
19	O43866	CD5 antigen-like (CD5L)	−1.19	0.00436	2.12e+08	9.68e+07	7.75e+07	2.65e+07

**Table 5:** Identification of differentially expressed proteins between the ischemic stroke and control groups.

No.	Accession	Protein name (symbol)	Fold change (Stroke/Control)	*P*-value	Control mean	Control SD	Ischemic stroke mean	Ischemic stroke SD
1	P69905	Hemoglobin subunit alpha (HBA)	3.49	0.000010	2.60e+07	1.08e+07	2.78e+08	1.41e+08
2	P68871	Hemoglobin subunit beta (HBB)	2.89	0.000006	1.05e+08	4.26e+07	7.60e+08	3.39e+08
3	P60709	Actin, cytoplasmic 1 (ACTB)	2.53	0.005081	1.85e+06	3.82e+05	1.05e+07	6.64e+06
4	P02042	Hemoglobin subunit delta (HBD)	2.52	0.006426	6.68e+05	3.83e+05	3.84e+06	2.53e+06
5	P00915	Carbonic anhydrase 1 (CA1)	2.13	0.007164	3.65E+05	1.36e+05	2.17e+06	1.65e+06
6	A0A286YFJ8	Immunoglobulin heavy constant gamma 4 (IGHG4)	1.66	0.000280	3.28e+08	1.16e+08	1.03e+09	4.00e+08
7	P13716	Delta-aminolevulinic acid dehydratase (ALAD)	1.64	0.001193	1.49E+05	7.38e+04	6.08e+05	4.63e+05
8	P04040	Catalase (CAT)	1.58	0.000326	1.30e+07	6.62e+06	3.29e+07	1.13e+07
9	P00738	Haptoglobin (HP)	1.2	0.007022	2.58e+08	1.01e+08	8.08e+08	6.47e+08
10	P20742	Pregnancy zone protein (PZP)	1.18	0.021412	1.30e+06	5.77e+05	3.04e+06	1.82e+06
11	A0A075B6H8	Probable non-functional immunoglobulin kappa variable 1D-42 (IGKV1D-42)	1.18	0.044303	1.61e+06	4.33e+05	3.20e+06	1.48e+06
12	A0A075B6S9	Probable non-functional immunoglobulin kappa variable 1–37 (IGKV1-37)	1.14	0.001213	2.99e+07	1.51e+07	5.12e+07	9.03e+06
13	A0A075B7D0	Immunoglobulin heavy variable 1/OR15-1 (IGHV1OR15–1)	1.02	0.034926	4.71e+07	9.41e+06	9.04e+07	3.94e+07
14	P80108	Phosphatidylinositol-glycan-specific phospholipase D (GPLD1)	1.02	0.022714	8.65e+05	1.91e+05	1.59e+06	6.14e+05
15	Q9BWP8	Collectin-11 (COLEC11)	−2.62	0.006341	3.85e+05	2.21e+05	8.97e+04	8.32e+04
16	P01880	Immunoglobulin heavy constant delta (IGHD)	−1.92	0.016525	3.50e+07	2.94e+07	8.05e+06	5.41e+06
17	O14791	Apolipoprotein L1 (APOL1)	−1.83	0.018448	2.66e+07	6.60e+06	1.18e+07	9.14e+06
18	P00739	Haptoglobin-related protein (HPR)	−1.52	0.048337	6.19e+07	2.56e+07	3.01e+07	2.48e+07
19	Q15848	Adiponectin (ADIPOQ)	−1.47	0.020377	6.79e+06	3.34e+06	3.29e+06	2.33e+06

**Figure 3:**
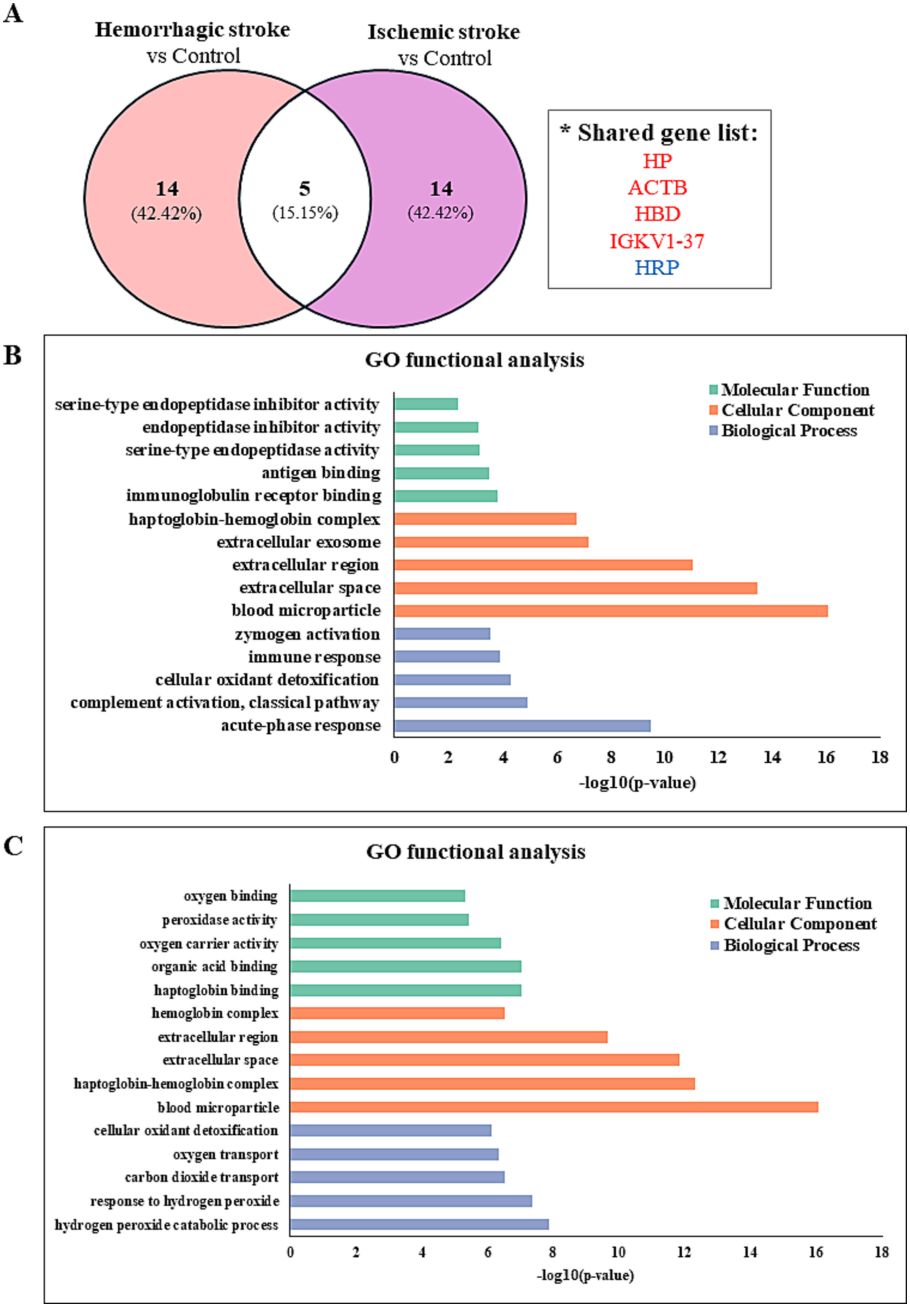
Identify the protein–protein interaction and gene ontology analyses in hemorrhagic and ischemic stroke patients. **(A)** Comparison of significantly differentially regulated proteins between HS and IS patients. Gene ontology enrichment analysis of proteins identified between **(B)** HS patients and controls and **(C)** IS patients and controls.

The proteomic profiles revealed unique protein alterations specific to each stroke subtype, underscoring the distinct pathophysiological mechanisms driving each condition (Figure 3A). Notably, five proteins exhibited consistent expression patterns across both HS and IS. Specifically, haptoglobin (HP), actin beta (ACTB), hemoglobin subunit delta (HBD), and probable non-functional immunoglobulin kappa variable 1–37 (IGKV1-37) were significantly upregulated, while haptoglobin-related protein (HRP) was notably downregulated in both conditions.

Functional enrichment analysis of DEPs was performed to further investigate their biological relevance. Gene ontology (GO) enrichment analysis was conducted using the DAVID database (version 2024q4). The 5 most significantly enriched terms (*p* < 0.05) in biological process, cellular component, and molecular function categories are presented in Figures 3B,C.

### 3.3 ELISA-based analysis of CRP and PF4 in HS patients

**Figure 4:**
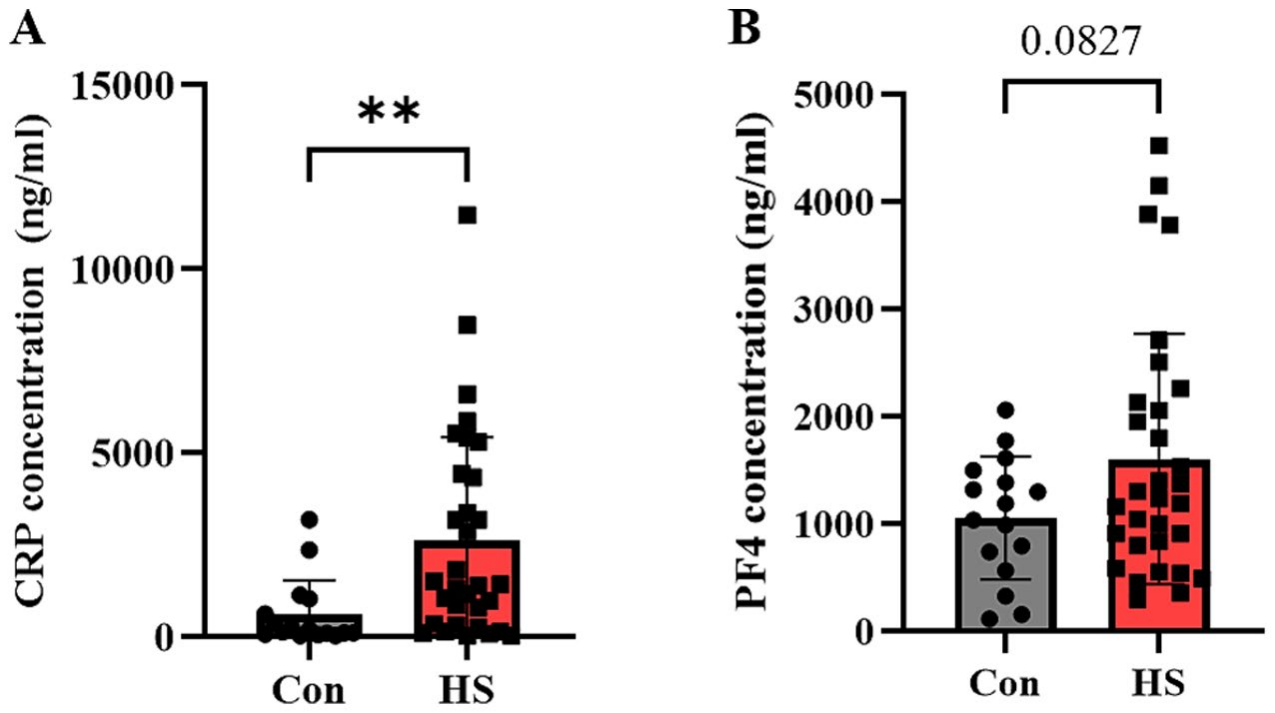
Quantification of protein markers in hemorrhagic stroke patients via ELISA. ELISA evaluated **(A)** CRP, **(B)** PF4 concentrations in human plasma. Individual data points are shown on bar graphs to visualize the distribution and variability within each group. CRP, C-reactive protein; PF4, Platelet factor 4. Con (*n* = 16), hemorrhagic stroke (*n* = 31). Statistical analysis was performed using a student’s *t*-test. Data were presented as mean ± SEM (**p < 0.01, ns = not significant).

Proteomic profiling of blood samples from HS patients revealed that CRP and PF4 not only increased substantially compared to controls but also displayed the most significant fold changes among all proteins analyzed (Table 4). Subsequently, we quantified these biomarkers across our study cohorts. As a result, CRP levels were substantially higher in the HS patient group (2571.0 ± 509.3 ng/mL) compared to the control group (620.7 ± 230.2 ng/mL) (Figure 4A; *p* = 0.0080). While PF4 levels were also elevated in the HS patient group (1603.7 ± 209.2 ng/mL) compared to the control group (1053.5 ± 573.1 ng/mL), this increase did not achieve statistical significance (Figure 4B; *p* = 0.0827). Nonetheless, the observed elevation in PF4 levels suggests a potential trend toward higher values in the HS group, indicating that both CRP and PF4 may serve as potential biomarkers for HS conditions.

## 4 Discussion

This preliminary study aimed to identify and analyze specific EV proteins that could potentially be stroke-associated biomarkers. To this end, we isolated EVs from the plasma of stroke patients (2.4 ± 1.0 days post-onset) and strictly healthy control individuals without major comorbidities, including those frequently associated with aging (e.g., hypertension, diabetes, obesity, or cardiovascular disease), and analyzed their expression profiles. We acknowledge that the age discrepancy between groups may have influenced the observed proteomic differences. However, recruiting elderly controls without such comorbidities was extremely challenging, as most individuals in that age group had one or more exclusionary conditions. Importantly, we intended to maximize biological contrast in this discovery phase, enabling the detection of strong stroke-associated signals that might otherwise be masked by overlapping age- or comorbidity-related alterations.

Both HS and IS shared the key pathological processes such as neuroinflammation, oxidative stress, and blood–brain barrier (BBB) dysfunction (10). These common mechanisms contribute to secondary neuronal injury and systemic inflammatory responses, which are reflected in EV protein profiles (11). In this study, the proteins, including ACTB, HP, HBD, and IGKV1-37 were commonly upregulated in EVs from both HS and IS patients. These proteins are indicative of key stroke-related pathological processes, including cellular damage, inflammation, and BBB disruption, aligning with findings from previous studies (12, 13). ACTB is a cytoskeletal protein that plays a critical role in maintaining cell shape and facilitating cellular movement. During cell necrosis or damage, intracellular components such as ACTB are released into the extracellular space, serving as direct evidence of cellular injury (14). To date, no studies have directly analyzed ACTB levels in stroke patients. However, increased ACTB expression has been reported in the injured brain tissue of traumatic brain injury mouse models (15). The increase in ACTB levels in EV from stroke patients may be linked to cytoskeletal remodeling, inflammatory responses, or stress conditions. Therefore, further research is needed to elucidate its biological significance. Recent studies suggest that HP plays a crucial protective role in reducing erythrocyte hemolysis and hemoglobin (Hb) toxicity in HS, particularly in intracerebral hemorrhage (ICH) (16). HP effectively binds to free Hb, mitigating oxidative stress and suppressing inflammation by preventing Hb auto-oxidation. Moreover, it limits the release of toxic heme and iron and prompts clearance via CD163-mediated endocytosis in macrophages (17). In ICH, vascular damage triggers erythrocyte destruction and subsequent Hb release, with elevated level of HP associated with further erythrocyte hemolysis and Hb release in the brain (18). Our proteomics analysis of EVs from both HS and IS patients revealed significantly elevated HP levels, suggesting its protective role in stroke pathology. Prior research indicated that in IS, reperfusion-induced oxidative stress and inflammation can damage erythrocytes and release Hb (19, 20). In contrast, HP plays a crucial role by neutralizing free Hb toxicity and reduce oxidative damage in HS (21). These findings suggest that HP may function as a compensatory mechanism to reduce damage caused by free Hb, including its associated oxidative stress and inflammation, within the brain. Although previous research has largely concentrated on the role of HP in HS, our findings, in conjunction with preceding studies, suggest that increased HP levels in IS may also have protective implications. However, a major limitation is that HP is primarily synthesized in the liver, producing about 30–50% of its total amount, and circulates through the bloodstream, resulting in relatively low concentrations in the cerebrospinal fluid (CSF) (22). As a hemoglobin-derived protein with a potential role as a subunit, HBD has not been directly implicated in stroke pathology in previous studies, but it may be associated with oxidative stress and inflammatory response regulation. The presence of hemoglobin-derived proteins in circulation may reflect the leakage of blood components following BBB disruption, contributing to increased inflammation and oxidative stress, which are linked to neuronal damage (23). Considering that hemoglobin degradation products such as heme and iron can induce secondary injury in stroke, HBD may also play a role in this pathological process (24). Therefore, further research is needed to determine whether the increased presence of HBD in EVs from stroke patients is merely a secondary phenomenon or if it plays a significant role in the pathological process. IGKV1-37 is a member of the immunoglobulin kappa light chain variable region family, specifically classified within the IGKV1 subfamily, and is predicted to be involved in immune responses. While immunoglobulin components are often considered contaminants in global plasma proteomics due to their abundance, the selective and significant elevation of IGKV1-37 in stroke patients—compared to healthy controls—suggests a biologically meaningful change. Although direct evidence linking IGKV1-37 to the pathogenesis of stroke is currently lacking, several studies have reported that members of the IGKV1 subfamily are associated with immune regulation and inflammatory responses (25, 26). Based on these findings, it is reasonable to hypothesize that IGKV1-37 may also be involved in the systemic immune response following stroke. Its elevated presence in plasma-derived exosomes may be indicative of stroke-associated acute inflammation and immune cell activation. It is also conceivable that IGKV1-37 is passively incorporated into exosomes released by activated immune cells as part of a generalized systemic response to neurovascular injury.

While HS and IS share common pathological mechanisms such as neuroinflammation, oxidative stress, and BBB dysfunction, the distinct pathological processes of HS and IS are likely to be reflected in the protein signatures of EV. HS occurs due to the rupture of blood vessels, leading to the leakage of blood into brain tissue. Key pathological features include hematoma formation, toxic responses in surrounding tissues, and subsequent inflammatory reactions (27). Our proteomic analysis identified proteins such as CRP, PF4, Inter-alpha-trypsin inhibitor heavy chain H3 (ITIH3), and Alpha-1-acid glycoprotein (ORM1) that are significantly upregulated in EVs from HS patients. These factors are involved in inflammation regulation, BBB repair, and neuroprotection, aligning with the pathological mechanisms underlying HS. CRP is an acute-phase reactant produced in the liver that increases in the blood when inflammation or tissue damage occurs, making it a sensitive indicator of the inflammatory state in the body. A 12-year prospective cohort study in Japan found no significant association between initial hsCRP levels and HS risk, while higher hsCRP levels in men were linked to increased IS incidence, suggesting its potential as a predictor for IS (28). In our study, we performed proteomic analysis of blood-derived EVs from patients, on average 1 to 3 days post-acute symptom onset, and found a significant upregulation of CRP in the HS group compared to the control group. Additionally, we observed a notable increase in plasma CRP levels in a separate cohort of HS patients. This aligns with previous findings that plasma CRP levels rise markedly within 48 to 72 h after intracerebral hemorrhage, correlating with hematoma volume and indicating its prognostic relevance. Furthermore, CRP has been detected at high levels around hemorrhagic lesions and in neurons and glial cells of patients who died within 12 h of hemorrhage (29). As such, CRP may play a role in predicting the prognosis or assessing outcomes in patients with acute post-hemorrhagic outcomes. PF4 is a protein primarily secreted by platelets, playing a crucial role in platelet aggregation and vascular activation. PF4 binds to anionic molecules to form immune complexes, which promote platelet activation and coagulation, leading to thrombosis and thrombocytopenia. Specifically, Vaccine-induced Immune Thrombotic Thrombocytopenia (VITT) and Spontaneous Heparin-Induced Thrombocytopenia (S-HIT) are characterized by the excessive inflammatory response driven by the formation of PF4-antibody complexes, which not only promote thrombosis but can also lead to severe complications such as intracerebral hemorrhage. Recent studies have reported frequent detection of PF4 antibodies in VITT patients (30), while S-HIT cases often show strong positivity for PF4 antibodies (31). These findings highlight the critical role of PF4 in the pathogenesis of VITT and S-HIT and suggest that similar mechanisms involving PF4 may play a significant role in patients with intracerebral hemorrhage. ITIH3 is a member of the inter-alpha-trypsin inhibitor protein family, which is mainly produced in the liver and found in plasma and extracellular matrix. It binds to hyaluronic acid to maintain the stability of the extracellular matrix and is involved in inflammatory responses and various physiological processes (32). Although no studies have directly linked it to hemorrhagic stroke, it has been implicated in human brain development (33), and the ITI family members perform a variety of physiological functions, including regulating inflammatory responses, inhibiting complement activation, and neutralizing extracellular histone toxicity (34), suggesting that it may play an important role in the pathological process of stroke. ORM1 has been identified as one of the differentially expressed genes in hypertensive cerebral hemorrhage, suggesting its potential involvement in the pathophysiology of hemorrhagic stroke (35). ORM1 has immune response modulation, vascular permeability regulation, and anti-inflammatory functions, and is likely involved in the regulation of inflammation and blood coagulation after HS, but the exact mechanisms require further studies including larger samples and *in vitro* and *in vivo* studies.

Ischemic stroke is primarily driven by ischemia–reperfusion injury as a key pathological mechanism. The reduction in cerebral blood flow during an ischemic stroke causes severe hypoxia, which in turn triggers a cascade of molecular responses aimed at restoring oxygen homeostasis (36). Proteomic analysis of EVs from IS patients revealed specific upregulation of proteins such as Hemoglobin subunit beta (HBB), Hemoglobin subunit alpha (HBA), Catalase (CAT), and Carbonic anhydrase 1 (CA1), which are associated with oxidative stress mitigation, oxygen transport, and pH regulation. Hemoglobin, primarily responsible for oxygen transport, plays a crucial role in maintaining cellular oxygenation. Specific studies report increased level of HBA and HBB in ischemic brain settings, suggesting an adaptive response to maintain mitochondrial and tissue homeostasis under oxygen-deprived environments (37). The upregulation of HBB and HBA in EVs suggests a compensatory cellular response to oxygen deprivation, potentially enhancing oxygen transport and delivery within ischemic brain tissue to mitigate hypoxic stress (38). Beyond their primary function in oxygen transport, hemoglobin subunits such as HBB and HBA are known to participate in oxidative stress regulation (39). Free hemoglobin can act as a double-edged sword: while it can scavenge reactive oxygen species (ROS) and reduce oxidative damage, it can also contribute to oxidative stress if heme is released and undergoes oxidation (40). The presence of HBB and HBA in EVs suggests a potential mechanism by which cells sequester hemoglobin to prevent excessive ROS accumulation in ischemic tissue, potentially serving as a protective response against oxidative damage and redox imbalance. Given that IS induces excessive production of reactive oxygen species, leading to a state of oxidative stress (36), the observed increase in CAT enrichment in EVs may represent a compensatory antioxidant response aimed at mitigating oxidative stress. Indeed, some studies propose leveraging the compensatory antioxidant role of CAT by enhancing its activity as a potential therapeutic strategy for stroke patients (41, 42). Several studies have investigated CAT levels in IS patients. Notably, a study evaluating serum CAT levels in acute ischemic stroke (AIS) patients over time reported a significant increase in catalase activity across all AIS groups compared to controls (43). Conversely, other studies have reported that CAT activity decreases during the acute phase of IS and has been interpreted as a suppressive effect induced by increased oxidative stress (44). Furthermore, CAT activity has been found to closely correlate with clinical outcomes in AIS patients, highlighting its potential role in stroke prognosis (45). Nevertheless, the aforementioned studies highlight the significant role of catalase in the pathophysiology of AIS, suggesting its potential as a therapeutic target and prognostic biomarker. Notably, catalase has been proposed as a potential oxidative stress biomarker for evaluating AIS prognosis, warranting further research to substantiate these findings. CA1 is a zinc metalloenzyme that catalyzes the hydration of carbon dioxide, the activation of which catalyzes the conversion of CO2 into bicarbonate ions (HCO3-) and protons (H+), thereby regulating pH (46). Additionally, the CA1 plays an important role in vascular function and blood flow regulation. Overexpression of CA1 can promote the secretion of inflammatory cytokines and vascular calcification, and in particular, in an ischemic stroke model, CA inhibitors increased cerebral blood flow and oxygen supply based on the cerebral vasodilation effect and significantly reduced neural tissue damage (47), suggesting that regulation of CA1 may be important for stroke treatment.

The EVs have garnered attention as potential biomarkers that reflect the pathophysiology of brain injury (48, 49), with the potential to improve diagnostic accuracy and guide therapeutic decision-making in clinical practice (50, 51). Considering their ability to encapsulate brain-derived molecular signatures, EVs were analyzed in this study as potential stroke biomarkers. Although our study provides preliminary insights into EV-derived biomarkers in stroke, it did not fully adhere to the Minimal Information for Studies of Extracellular Vesicles (MISEV) guidelines. While these guidelines recommend comprehensive EV isolation, characterization, and functional validation standards, the current study focused on EV proteomic profiling using clinical samples obtained under practical constraints. Due to limited sample volume, we were unable to perform further characterization steps such as EV surface marker profiling or electron microscopy. However, we characterized EV size using NTA and followed a standardized commercial isolation protocol (ExoQuick™), which has been widely used in clinical EV research. We acknowledge that a more rigorous application of MISEV guidelines would strengthen the robustness and reproducibility of EV-based biomarker studies. Future work will aim to incorporate these guidelines more comprehensively, including additional EV markers and orthogonal validation techniques, particularly in follow-up studies with expanded sample cohorts.

Given the potential clinical applications of biomarker derived from EVs, we performed follow-up ELISA-based experiments on these biomarkers using plasma samples. Plasma provides a methodologically appropriate approach due to its simplified sample preparation and higher extraction efficiency, which is advantageous for handling large-scale samples (52). Although EVs-derived biomarkers hold potential as clinical diagnostic tools, their application is currently limited because EV isolation and analysis are restricted to specific research settings (53). In contrast, plasma is already an easily accessible and analyzable biological sample in clinical practice. While standardized EV collection and analysis methods are still under debate (54), the development of a standardized approach specific to stroke patients in the future could enable the implementation of patient-tailored diagnostics. Given these challenges, plasma biomarkers remain a more immediately accessible alternative for clinical application. Therefore, we performed ELISA-based analysis using plasma samples, focusing on CRP and PF4, which showed the most prominent increases in our proteomic dataset. Due to limited sample availability, we prioritized these top candidates rather than conducting a broad validation across all differentially expressed proteins. We acknowledge this as a limitation and plan to include additional targets and a larger validation cohort in future studies to assess their diagnostic and clinical utility further. In this preliminary study, ELISA was conducted only in HS samples due to limitations in securing a sufficient number of IS samples. We acknowledge this as a limitation and plan to include IS samples in future validations. Overall, analyzing candidate biomarkers in plasma represents a crucial step toward facilitating clinical translation, requiring further validation in larger cohorts and large-scale studies (55).

In conclusion, this pilot study investigated the pathological differences in specific biomarkers derived from EVs in patients with HS and IS compared to controls. Although it did not directly elucidate whether these biomarkers have neuronal specificity or are specifically associated with the affected lesion site, it evaluated the potential of EVs as biomarkers by identifying DEPs in blood-derived EVs from different stroke types. Importantly, this study includes an analysis of these biomarkers in the blood of HS patients, supporting their clinical applicability for stroke prediction and treatment. The biomarkers that showed significant increases in plasma-derived EVs but not in plasma may be due to the selective release and enrichment of biomarkers in EVs, whereas in plasma, they may be diluted or interact with other proteins, reducing detection sensitivity. Additionally, unlike proteins protected within EVs, those in plasma exist in free form or may undergo modifications, potentially leading to non-significant differences. Therefore, future studies should systematically investigate the differences in biomarker expression between EVs and plasma. Additionally, clarifying the cellular origin and functional roles of EVs, as well as examining biomarker variations across disease stages and individual patient characteristics, will be crucial for strengthening the link between EV-derived and plasma-based biomarkers, ultimately improving their clinical utility.

## Data Availability

The proteomic datasets generated and analyzed for this study have been uploaded in the ProteomeXchange Consortium via the PRIDE partner repository under accession number PXD062958, and are available at the following URL: https://www.ebi.ac.uk/pride/archive/projects/PXD062958.
