# Correction: Identification of stroke biomarkers using proteomic profiling of extracellular vesicles derived from human blood: a preliminary study

**DOI:** 10.3389/fneur.2025.1736367

**Published:** 2025-11-24

**Authors:** Tae Yeon Kim, Jae Yeon Park, Yun Seo Cho, Dong Hyuk Youn, Sung Woo Han, Harry Jung, Yong-Jun Cho, In cheol Jeong, Jin Pyeong Jeon, Kangeun Ko, Hyo Youl Moon

**Affiliations:** 1 Department of Physical Education, Seoul National University, Seoul, Republic of Korea; 2 Institute of New Frontier Research, Hallym University College of Medicine, Chuncheon, Republic of Korea; 3 Department of Neurosurgery, Hallym University College of Medicine, Chuncheon, Republic of Korea; 4 Department of Artificial Intelligence Convergence, Hallym University, Chuncheon, Republic of Korea; 5 Institute of Aging, Seoul National University, Seoul, Republic of Korea; 6 Learning Sciences Research Institute, Seoul National University, Seoul, Republic of Korea

**Keywords:** hemorrhagic stroke, ischemic stroke, extracellular vesicles, biomarker, proteomics

In the published article, an error appeared in the abstract. The sentence stated:

Among these, 14 proteins were commonly upregulated and 1 protein was commonly downregulated in both stroke types, whereas the remaining proteins exhibited stroke-type-specific expression patterns.

This has been corrected to read:

Among these, four proteins were commonly upregulated and one protein was commonly downregulated in both stroke types, whereas the remaining proteins exhibited stroke-type-specific expression patterns.

A correction is also required to the section **Discussion**, Paragraph 3:

Furthermore, CRP has been detected at high levels around hemorrhagic lesions and in neurons and glial cells of patients who died within 12 h of hemorrhage (“*C-Reactive Protein in Intracerebral Hemorrhage Time Course, Tissue Localization, and Prognosis Supplemental Data at www.Neurology.Org*”* 2012*) (29).

The updated sentence appears below:

Furthermore, CRP has been detected at high levels around hemorrhagic lesions and in neurons and glial cells of patients who died within 12 h of hemorrhage (29).

The original version of this article has been updated.

